# 1-Allyl-1*H*-1,3-benzimidazol-2(3*H*)-one

**DOI:** 10.1107/S1600536812043620

**Published:** 2012-10-27

**Authors:** Dounia Belaziz, Youssef Kandri Rodi, Fouad Ouazzani Chahdi, El Mokhtar Essassi, Mohamed Saadi, Lahcen El Ammari

**Affiliations:** a Laboratoire de Chimie Organique Appliquée, Université Sidi Mohamed Ben Abdallah, Faculté des Sciences et Techniques, Route d’immouzzer, BP 2202 Fès, Morocco; b Laboratoire de Chimie Organique Hétérocyclique URAC21, Faculté des Sciences, Université Mohammed V-Agdal, Avenue Ibn Battouta, BP 1014, Rabat, Morocco; c Institute of Nanmaterials and Nanotechnology, MASCIR, Rabat, Morocco; d Laboratoire de Chimie du Solide Appliquée, Faculté des Sciences, Université Mohammed V-Agdal, Avenue Ibn Battouta, BP 1014, Rabat, Morocco

## Abstract

The fused five- and six-membered rings in the title compound, C_10_H_10_N_2_O, are approximately coplanar, with an r.m.s. deviation of 0.008 Å. The mean plane of the allyl group is roughly perpendicular to the mean plane of the 1,3-benzimidazol-2(3*H*)-one system, making a dihedral angle of 86.1 (2)°. In the crystal, each mol­ecule is linked to its symmetry equivalent partner by a pair of N—H⋯O and C—H⋯O hydrogen bonds.

## Related literature

For the pharmacological and biochemical properties of the title compound, see: Gravatt *et al.* (1994 ▶); Horton *et al.* (2003 ▶); Kim *et al.* (1996 ▶); Roth *et al.* (1997 ▶). For compounds with similar structures, see: Belaziz *et al.* (2012 ▶); Ouzidan *et al.* (2011 ▶).
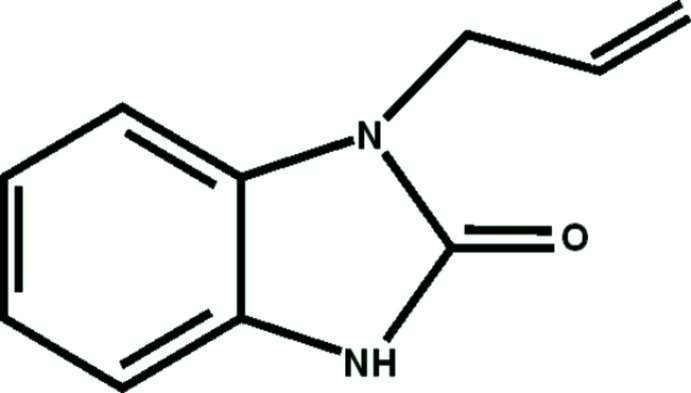


## Experimental

### 

#### Crystal data

C_10_H_10_N_2_O
*M* *_r_* = 174.20
Monoclinic, 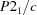

*a* = 10.2749 (5) Å
*b* = 5.5787 (3) Å
*c* = 16.6220 (9) Å
β = 100.976 (4)°
*V* = 935.35 (8) Å^3^
*Z* = 4
Mo *K*α radiation
μ = 0.08 mm^−1^
*T* = 296 K
0.38 × 0.29 × 0.27 mm

#### Data collection

Bruker X8 APEX diffractometer
13429 measured reflections
2570 independent reflections
1393 reflections with *I* > 2σ(*I*)
*R*_int_ = 0.046

#### Refinement

*R*[*F* ^2^ > 2σ(*F* ^2^)] = 0.045
*wR*(*F* ^2^) = 0.128
*S* = 1.04
2570 reflections
120 parameters
H-atom parameters constrained
Δρ_max_ = 0.14 e Å^−3^
Δρ_min_ = −0.14 e Å^−3^

### 

Data collection: *APEX2* (Bruker, 2005 ▶); cell refinement: *SAINT* (Bruker, 2005 ▶); data reduction: *SAINT*; program(s) used to solve structure: *SHELXS97* (Sheldrick, 2008 ▶); program(s) used to refine structure: *SHELXL97* (Sheldrick, 2008 ▶); molecular graphics: *ORTEP-3 for Windows* (Farrugia, 2012 ▶); software used to prepare material for publication: *PLATON* (Spek, 2009 ▶) and *publCIF* (Westrip, 2010 ▶).

## Supplementary Material

Crystal structure: contains datablock(s) I, global. DOI: 10.1107/S1600536812043620/fj2602sup1.cif

Structure factors: contains datablock(s) I. DOI: 10.1107/S1600536812043620/fj2602Isup2.hkl

Supplementary material file. DOI: 10.1107/S1600536812043620/fj2602Isup3.cml

Additional supplementary materials:  crystallographic information; 3D view; checkCIF report

## Figures and Tables

**Table 1:** Hydrogen-bond geometry (Å, °)

*D*—H⋯*A*	*D*—H	H⋯*A*	*D*⋯*A*	*D*—H⋯*A*
N2—H2⋯O1^i^	0.86	2.00	2.8274 (14)	161
C3—H3⋯O1^ii^	0.93	2.52	3.3080 (19)	142

Symmetry codes: (i) 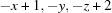; (ii) 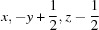.

**Fig. 1.**
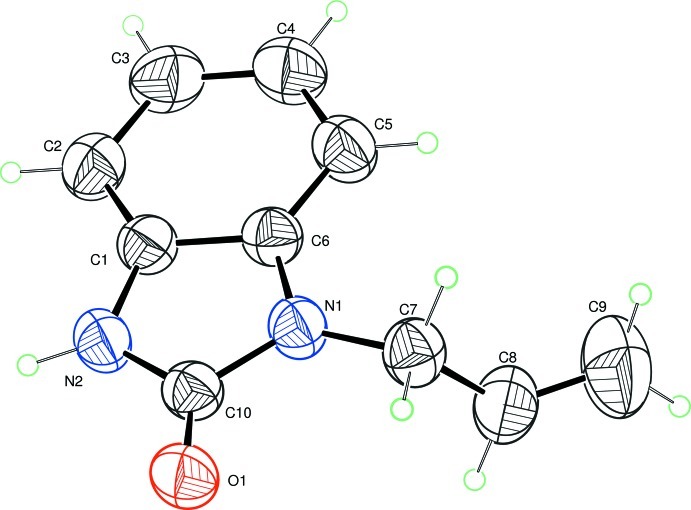
Molecular structure of the title compound with the atom-labelling scheme. Displacement ellipsoids are drawn at the 50% probability level. H atoms are represented as small circles.

**Fig. 2.**
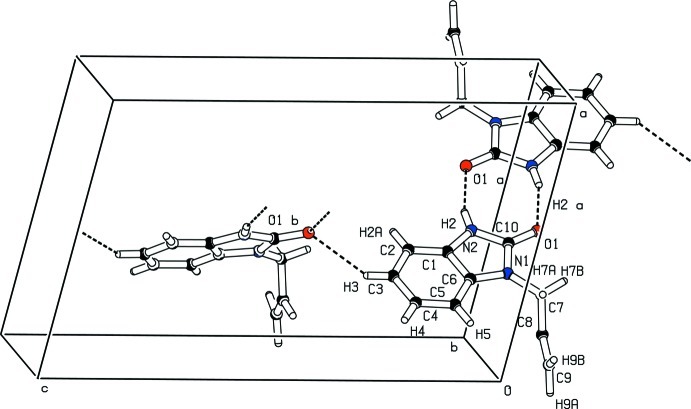
Molecule and its symmetry through the inversion center linked by hydrogen bonds and building dimers.

## supplementary crystallographic information

### Comment

Benzimidazoles are very useful intermediates/subunits for the development of molecules of pharmaceutical or biological interest. Benzimidazole and its derivatives are an important class of bioactive molecules in the field of drugs and pharmaceuticals. Benzimidazole derivatives have found applications in diverse therapeutic areas including anti-ulcers, anti-hypertensives, anti-virals, anti-fungals, anti-cancers, (Gravatt *et al.* 1994; Horton *et al.* 2003; Kim *et al.* 1996; Roth *et al.* 1997).

As a continuation of our research work devoted to the development of substituted benzimidazol-2-one derivatives (Belaziz *et al.*, 2012; Ouzidan *et al.* 2011), we reported in this paper the synthesis of new benzimidazol-2-one derivative by action of allyl bromide with 1*H*-1,3-benzimidazol-2(3*H*)-one in the presence of a catalytic quantity of tetra-n-butylammonium bromide under mild conditions to furnish mono-substituted compound (Scheme 1).

The crystal structure of the 1-allyl-1*H*-1,3-benzimidazol-2(3*H*)-one molecule is built up from fused six-and five-membered rings linked to C_3_H_5_ chain as shown in Fg.1. The fused-ring system is essentially planar, with a maximum deviation of -0.010 (1) Å for C10. The allyl group is nearly perpendicular to the 1*H*-1,3-benzimidazol-2(3*H*)-one plane as indicated by the torsion angle of C8 C7 N1 C6 - 70.44 (18)°. In the crystal, each molecule and its symmetry through the inversion center are linked by N2—H2···O1 and C3—H3···O1 hydrogen bonds in the way to form dimers as shown in Fig.2.

### Experimental

To 1*H*-1,3-benzimidazol-2(3*H*)-one (0.2 g, 1.49 mmol), potassium carbonate (0.41 g, 2.98 mmol) and tetra-n-butylammonium bromide (0.05 g, 0.15 mmol) in DMF (15 ml) was added allyl bromide (0.14 ml, 1.78 mmol). Stirring was continued at room temperature for 6 h. The salt was removed by filtration and the filtrate concentrated under reduced pressure. The residue was separated by chromatography on a column of silica gel with ethyl acetate/hexane (1/2) as eluent. The product was obtained with quantitative yield of 70%. It was recrystallized from hexan/acetate to give colourless crystals.

### Refinement

H atoms were located in a difference map and treated as riding with N—H = 0.86 Å, C—H = 0.93 Å (aromatic), and C—H = 0.97 Å (methylene). with *U*_iso_(H) = 1.2 *U*_eq_ (aromatic, methylene).

### Crystal data

**Table:**

C_10_H_10_N_2_O	*F*(000) = 368
*M**_r_* = 174.20	*D*_x_ = 1.237 Mg m^−^^3^
Monoclinic, *P*2_1_/*c*	Melting point: 342.7 K
Hall symbol: -p 2ybc	Mo *K*α radiation, λ = 0.71073 Å
*a* = 10.2749 (5) Å	Cell parameters from 2570 reflections
*b* = 5.5787 (3) Å	θ = 2.9–29.4°
*c* = 16.6220 (9) Å	µ = 0.08 mm^−^^1^
β = 100.976 (4)°	*T* = 296 K
*V* = 935.35 (8) Å^3^	Block, colourless
*Z* = 4	0.38 × 0.29 × 0.27 mm

### Data collection

**Table:**

Bruker X8 APEX diffractometer	1393 reflections with *I* > 2σ(*I*)
Radiation source: fine-focus sealed tube	*R*_int_ = 0.046
Graphite monochromator	θ_max_ = 29.4°, θ_min_ = 2.9°
φ and ω scans	*h* = −13→14
13429 measured reflections	*k* = −7→7
2570 independent reflections	*l* = −22→22

### Refinement

**Table:**

Refinement on *F*^2^	Secondary atom site location: difference Fourier map
Least-squares matrix: full	Hydrogen site location: difference Fourier map
*R*[*F*^2^ > 2σ(*F*^2^)] = 0.045	H-atom parameters constrained
*wR*(*F*^2^) = 0.128	*w* = 1/[σ^2^(*F*_o_^2^) + (0.0584*P*)^2^] where *P* = (*F*_o_^2^ + 2*F*_c_^2^)/3
*S* = 1.04	(Δ/σ)_max_ < 0.001
2570 reflections	Δρ_max_ = 0.14 e Å^−^^3^
120 parameters	Δρ_min_ = −0.14 e Å^−^^3^
0 restraints	Extinction correction: *SHELXL97* (Sheldrick, 2008), Fc^*^=kFc[1+0.001xFc^2^λ^3^/sin(2θ)]^-1/4^
Primary atom site location: structure-invariant direct methods	Extinction coefficient: 0.011 (4)

### Special details

**Table:**

Geometry. All s.u.'s (except the s.u. in the dihedral angle between two l.s. planes) are estimated using the full covariance matrix. The cell s.u.'s are taken into account individually in the estimation of s.u.'s in distances, angles and torsion angles; correlations between s.u.'s in cell parameters are only used when they are defined by crystal symmetry. An approximate (isotropic) treatment of cell s.u.'s is used for estimating s.u.'s involving l.s. planes.
Refinement. Refinement of *F*^2^ against ALL reflections. The weighted *R*-factor *wR* and goodness of fit *S* are based on *F*^2^, conventional *R*-factors *R* are based on *F*, with *F* set to zero for negative *F*^2^. The threshold expression of *F*^2^ > 2σ(*F*^2^) is used only for calculating *R*-factors(gt) *etc*. and is not relevant to the choice of reflections for refinement. *R*-factors based on *F*^2^ are statistically about twice as large as those based on *F*, and *R*- factors based on ALL data will be even larger.

### Fractional atomic coordinates and isotropic or equivalent isotropic displacement parameters (Å^2^)

**Table:**

	*x*	*y*	*z*	*U*_iso_*/*U*_eq_	
C1	0.64101 (14)	0.3295 (2)	0.87918 (9)	0.0444 (4)	
C2	0.63854 (16)	0.3098 (3)	0.79675 (9)	0.0549 (4)	
H2A	0.5937	0.1851	0.7662	0.066*	
C3	0.70534 (18)	0.4825 (3)	0.76048 (9)	0.0638 (5)	
H3	0.7056	0.4732	0.7046	0.077*	
C4	0.77162 (19)	0.6684 (3)	0.80584 (10)	0.0651 (5)	
H4	0.8153	0.7820	0.7798	0.078*	
C5	0.77454 (16)	0.6892 (2)	0.88889 (10)	0.0557 (4)	
H5	0.8189	0.8149	0.9191	0.067*	
C6	0.70925 (14)	0.5166 (2)	0.92520 (8)	0.0436 (4)	
C7	0.74323 (15)	0.6384 (2)	1.07492 (9)	0.0512 (4)	
H7A	0.7020	0.5928	1.1205	0.061*	
H7B	0.7180	0.8027	1.0605	0.061*	
C8	0.88971 (17)	0.6264 (3)	1.10148 (10)	0.0656 (5)	
H8	0.9191	0.4602	1.1144	0.105 (7)*	
C9	0.9688 (2)	0.8074 (4)	1.11471 (13)	0.0968 (7)	
H9A	1.0714	0.7945	1.1346	0.116*	
H9B	0.9256	0.9702	1.1048	0.116*	
C10	0.61611 (15)	0.2860 (2)	1.01062 (9)	0.0441 (4)	
N1	0.69312 (11)	0.48505 (18)	1.00561 (7)	0.0452 (3)	
N2	0.58638 (12)	0.19162 (19)	0.93391 (7)	0.0481 (3)	
H2	0.5401	0.0640	0.9210	0.058*	
O1	0.58255 (11)	0.21105 (17)	1.07330 (6)	0.0559 (3)	

### Atomic displacement parameters (Å^2^)

**Table:**

	*U*^11^	*U*^22^	*U*^33^	*U*^12^	*U*^13^	*U*^23^
C1	0.0415 (9)	0.0445 (7)	0.0468 (9)	0.0002 (5)	0.0072 (7)	0.0025 (6)
C2	0.0578 (11)	0.0578 (9)	0.0476 (10)	−0.0040 (7)	0.0064 (8)	−0.0033 (6)
C3	0.0733 (13)	0.0750 (11)	0.0442 (9)	−0.0033 (8)	0.0137 (8)	0.0056 (7)
C4	0.0751 (13)	0.0680 (11)	0.0555 (11)	−0.0120 (8)	0.0205 (9)	0.0106 (8)
C5	0.0597 (11)	0.0523 (9)	0.0567 (10)	−0.0101 (7)	0.0149 (8)	0.0027 (6)
C6	0.0417 (9)	0.0443 (7)	0.0451 (8)	0.0006 (6)	0.0091 (6)	0.0028 (5)
C7	0.0556 (11)	0.0511 (8)	0.0483 (9)	−0.0034 (6)	0.0132 (8)	−0.0068 (6)
C8	0.0591 (12)	0.0633 (11)	0.0695 (12)	−0.0024 (8)	0.0001 (9)	−0.0102 (8)
C9	0.0674 (15)	0.0902 (15)	0.130 (2)	−0.0206 (10)	0.0128 (13)	−0.0189 (12)
C10	0.0420 (9)	0.0437 (7)	0.0469 (9)	0.0000 (6)	0.0090 (7)	0.0037 (6)
N1	0.0476 (8)	0.0444 (6)	0.0442 (7)	−0.0063 (5)	0.0106 (6)	−0.0014 (4)
N2	0.0514 (8)	0.0439 (6)	0.0491 (8)	−0.0094 (5)	0.0100 (6)	−0.0008 (5)
O1	0.0631 (8)	0.0575 (6)	0.0491 (7)	−0.0111 (5)	0.0159 (6)	0.0066 (4)

### Geometric parameters (Å, º)

**Table:**

C1—C2	1.370 (2)	C7—N1	1.4487 (17)
C1—N2	1.3890 (17)	C7—C8	1.487 (2)
C1—C6	1.4007 (18)	C7—H7A	0.9700
C2—C3	1.386 (2)	C7—H7B	0.9700
C2—H2A	0.9300	C8—C9	1.288 (2)
C3—C4	1.383 (2)	C8—H8	0.9858
C3—H3	0.9300	C9—H9A	1.0463
C4—C5	1.380 (2)	C9—H9B	1.0104
C4—H4	0.9300	C10—O1	1.2313 (16)
C5—C6	1.3768 (19)	C10—N2	1.3594 (17)
C5—H5	0.9300	C10—N1	1.3751 (17)
C6—N1	1.3890 (16)	N2—H2	0.8600
			
C2—C1—N2	132.67 (13)	C8—C7—H7A	108.9
C2—C1—C6	121.20 (13)	N1—C7—H7B	108.9
N2—C1—C6	106.13 (12)	C8—C7—H7B	108.9
C1—C2—C3	117.58 (14)	H7A—C7—H7B	107.7
C1—C2—H2A	121.2	C9—C8—C7	125.79 (19)
C3—C2—H2A	121.2	C9—C8—H8	122.9
C4—C3—C2	121.15 (15)	C7—C8—H8	111.0
C4—C3—H3	119.4	C8—C9—H9A	124.4
C2—C3—H3	119.4	C8—C9—H9B	115.7
C5—C4—C3	121.56 (14)	H9A—C9—H9B	119.9
C5—C4—H4	119.2	O1—C10—N2	127.79 (13)
C3—C4—H4	119.2	O1—C10—N1	125.63 (13)
C6—C5—C4	117.39 (14)	N2—C10—N1	106.57 (12)
C6—C5—H5	121.3	C10—N1—C6	109.64 (11)
C4—C5—H5	121.3	C10—N1—C7	123.39 (12)
C5—C6—N1	131.91 (13)	C6—N1—C7	126.93 (11)
C5—C6—C1	121.11 (13)	C10—N2—C1	110.67 (12)
N1—C6—C1	106.97 (11)	C10—N2—H2	124.7
N1—C7—C8	113.31 (12)	C1—N2—H2	124.7
N1—C7—H7A	108.9		
			
N2—C1—C2—C3	−179.87 (15)	N2—C10—N1—C6	1.06 (15)
C6—C1—C2—C3	−0.4 (2)	O1—C10—N1—C7	−1.5 (2)
C1—C2—C3—C4	−0.2 (3)	N2—C10—N1—C7	178.85 (12)
C2—C3—C4—C5	0.3 (3)	C5—C6—N1—C10	178.59 (15)
C3—C4—C5—C6	0.3 (3)	C1—C6—N1—C10	−0.59 (15)
C4—C5—C6—N1	179.99 (14)	C5—C6—N1—C7	0.9 (2)
C4—C5—C6—C1	−0.9 (2)	C1—C6—N1—C7	−178.28 (13)
C2—C1—C6—C5	1.0 (2)	C8—C7—N1—C10	112.16 (16)
N2—C1—C6—C5	−179.39 (13)	C8—C7—N1—C6	−70.44 (18)
C2—C1—C6—N1	−179.73 (12)	O1—C10—N2—C1	179.19 (14)
N2—C1—C6—N1	−0.11 (15)	N1—C10—N2—C1	−1.14 (15)
N1—C7—C8—C9	130.56 (19)	C2—C1—N2—C10	−179.66 (15)
O1—C10—N1—C6	−179.26 (13)	C6—C1—N2—C10	0.79 (16)

### Hydrogen-bond geometry (Å, º)

**Table:**

*D*—H···*A*	*D*—H	H···*A*	*D*···*A*	*D*—H···*A*
N2—H2···O1^i^	0.86	2.00	2.8274 (14)	161
C3—H3···O1^ii^	0.93	2.52	3.3080 (19)	142

Symmetry codes: (i) −*x*+1, −*y*, −*z*+2; (ii) *x*, −*y*+1/2, *z*−1/2.
